# Dismissing the idea that basketball is a “contactless” sport: quantifying contacts during professional gameplay

**DOI:** 10.3389/fspor.2024.1419088

**Published:** 2024-07-23

**Authors:** Dennis Wellm, Johannes Jäger, Karen Zentgraf

**Affiliations:** ^1^ Institute of Sport Sciences, Department of Movement Science and Training in Sports, Goethe University, Frankfurt am Main, Germany; ^2^ Institute of Sport Science and Sport, Department of Sport and Exercise Medicine, Friedrich-Alexander-University, Erlangen, Germany

**Keywords:** monitoring, physical contact, basketball, load management, game analysis

## Abstract

**Introduction:**

Basketball, introduced by Naismith as a contactless and indoor alternative to sports such as American football, now frequently involves physical contact among players, challenging the traditional notion. Up to date, a thorough understanding of these contacts and their implications remains limited. This study aims to analyze player contacts, embedding it within overall load monitoring to optimize performance and reduce injury risk.

**Methods:**

Using a mixed-method design, video-based observations and quantitative analysis were employed to study contact characteristics during ten professional male basketball matches. Fisher exact tests and chi-squared tests (*p* < .05) were conducted to examine positional variations across different contact variables.

**Results:**

A total of 2,069 player contacts were examined, showing centers had the most contacts at 40.5%, followed by power forwards (19.6%), point guards (17.7%), shooting guards (12.9%), and small forwards (9.3%). Notably, half-court defense (46.1%) and set offense (48.9%) emerged as the primary game phases associated with the majority of contacts across all playing positions. Key play actions leading to physical contact included screening/picking (25.7%), box outs (22.9%), and fights for position (FFP) (18%). *Post hoc* analyses identified significant associations between centers (32.6%, 5.93) and point guards (21.5%, −1.98) during screening/picking maneuvers. Moreover, the torso/upper body (48.1%) and upper extremities (38.2%) were identified as the most affected contact points, while lower extremities and the head/neck exhibited minimal impact. Additionally, 81.4% (*n* = 1,684) of contacts resulted in kinematic displacement, whereas 18.6% (*n* = 385) exhibited no change. *Post hoc* analyses indicated significant associations of physical contacts against opposing counterparts for each playing position.

**Discussion:**

Basketball entails frequent physical contacts across all playing positions, with distinct patterns observed for each playing position. Integrating contact monitoring alongside traditional load metrics offers a more comprehensive understanding of physical demands in professional basketball. Practical implications include the developing of tailored training strategies based on playing position-specific contact profiles and recognizing the physiological and biomechanical impacts of contacts. Future research should consider whether the number of contacts between players has increased over the years, and it should acknowledge the impact of player contacts on performance in basketball in order to refine training strategies and enhance player well-being.

## 1 Introduction

Basketball was introduced in 1891 as an alternative physical activity to traditional sports such as American football or baseball (1). It is an intermittent sport characterized by the physical demands of requiring players to execute repeated high-intensity actions (2, 3). These actions include rapid changes of direction, jumping, cutting, and high-speed movements over short distances (2, 4, 5). Coaches and staff implement training strategies aiming to enhancing players' performance on the court. Furthermore, training periodization is utilized to monitor and manage players' fatigue levels (4, 6). Accordingly, load monitoring plays a pivotal role in furnishing valuable information for the development of training programs and maximizing physical performance while preventing overreaching and reducing injury risk (7–9). Monitoring external and internal loads, both during training and competition, is acknowledged as being crucial for athlete management across different training and competition phases (10–15). Moreover, it is also important to understand the extent to which players are exposed to game-like demands during practice sessions (16–20).

In basketball, the most widely used external load tracking metrics are currently total distance, relative distance (distance/duration), time in speed zones (e.g., total, relative and percentages), high-intensity actions (e.g., time and counts of accelerations, decelerations, jumps, player load metrics), and peak velocity (, 2, 5, 13, 20 ). These are usually measured with high validity using local positioning systems (LPS) (21), global positioning systems (GPS) (22), video-based time-motion analysis (TMA) (10, 23), or inertial movement analysis (IMA) including tri-axial accelerometry, gyroscope, and magnetometer data (13, 20, 24, 25). External load variables are useful when considering the role of contextual factors such as gender (26), team quality (27), playing position (8, 28, 29), ball possession status (30), game period (3, 31, 32), game outcome (33), final score differences (per period) (31), and accumulated point differences (per period) (31). Ultimately, the external load (e.g., accelerations) influences the degree of internal load (e.g., cardiovascular or metabolic) that represent the psychobiological response to the stimuli imposed by physical practice and game demands (34).

While monitoring the external load on basketball players is now common practice, the contribution of physical contacts to the external–internal load relationship is limited. Rice (35) emphasizes that athletes routinely make contact with each other in basketball, but usually with less force than in typical collision sports such as rugby. Besides the running demands, players frequently engage in quick and forceful physical interactions during key phases of offensive and defensive possession. Thus, repeated physical contacts are fundamental in basketball (3, 8, 36, 37). For example, when a player posts up, they use their body to establish position close to the basket, often against a defender who is trying to push them away. Similarly, when setting or fighting through screens, players must withstand and apply considerable force to create or prevent scoring opportunities (8). Also, the importance of boxing out has been a key performance indicator during rebounding (38). These common play actions during rebounding scenarios involve intense physical contact, as players use their bodies to gain advantageous positioning over opponents.

To date, analyses of contact events in basketball have been largely restricted, with limited information on the frequency and context of impact events. García et al. (32) noted that guards are typically less involved in scenarios involving high-impact body contact with opponents compared to forwards and centers. In addition, Johnston et al. (39), focusing on physical contacts during small-sided rugby games reported that contact in game-based activities induces more upper-body neuromuscular fatigue, a greater and longer lasting increase in plasma creatine kinase activity, and an increased perception of effort than game-based activities involving no contact. Importantly, for a comprehensive understanding of overall training load, valid measurements of both the volume and intensity of contacts are essential, because these actions provide a greater subjective, physical, and physiological load than noncontact training or high-intensity intermittent running alone (39).

The quantification of contacts and their significance for the external–internal load relationship provide valuable information about the physical demands of the playing positions (40). Guards, for instance, are primarily involved in accelerative and decelerative scenarios, such as perimeter play and one-on-one attacks, which generally involve less physical contact but require higher peak velocities compared to other positions (32). Forwards, on the other hand, engage less in high-intensity actions (41) and are frequently involved in physical battles for rebounds and screens, leading to more instances of body contact (3). Centers often experience the most physical contact as they are typically involved in posting up, boxing out, and protecting the rim (42). Due to tactical principles, centers usually occupy smaller court dimensions around the basket (13), resulting in the lowest total distance covered during matches (32). Conversely, Ferioli et al. (8) and Svilar et al. (43) found that centers exhibited the highest number of high-intensity accelerations, jumps, and high-intensity specific movements during training sessions and seasonal games, emphasizing a variance in positional requirements across training and competition modes (32). The quantification of contact loads (e.g., screens, box outs, post ups) alongside more traditional running metrics (e.g., distances covered) and high-intensity efforts (e.g., accelerations) would offer a more comprehensive picture of the external and internal demands of basketball by considering different playing positions (8, 31–33). For the best of the authors' knowledge, no previous research has been published related specifically to the quantification of physical contacts and contextual variables in professional basketball.

Therefore, the primary objective of this study is to observe and quantify contacts during professional male basketball matches and to analyze how contact situations are distributed across playing positions. Positional contacts are expected to demonstrate dependencies on situational patterns such as games phase, play action, and the opponent's playing position. By examining the occurrence of contacts, the goal of this study is to enhance the understanding of individual activity profiles and playing positions in the context of athlete monitoring in basketball. Additionally, the study aims to highlight the distinct demands associated with each playing position and provide practical applications for training purposes.

## 2 Materials and methods

### 2.1 Experimental design

Following Creswell and Plano Clark's (44) methodology, this study employed a predetermined fixed mixed-method design. This systematic approach integrates qualitative and quantitative data, with both components predetermined at the beginning of the research process. At first, qualitative data collection enables visual inspection and initial description. Subsequently, a quantitative summary, guided by standardized observational elements, complements prior qualitative insights, aiming to support the overall qualitative estimation. Synthesizing and controlling the data are followed by a conclusive qualitative stage. In this phase, results are presented and interpreted in the context of the previously identified research problem. Merging qualitative and quantitative components establishes methodological symmetry, thereby fostering a comprehensive approach that is deemed advantageous for drawing final conclusions (45). This interconnection ensures a holistic perspective, promoting a more nuanced and well-rounded understanding of the research phenomena.

### 2.2 Participants

A total of 19 players from one team were included in the analyzed cohort over the study period. Players were categorized into five positional groups (defined by the head coach): point guard (PG) (*n* = 8, mean age = 23.1 ± 4.6 years, mean height = 190 ± 0.1 cm, mean weight = 82.6 ± 8.1 kg), shooting guard (SG) (*n* = 3, mean age, 28.5 ± 8 years, mean height = 1.93 ± 0.3 cm, mean weight = 90.7 ± 5.9 kg), small forward (SF) (*n* = 2, mean age, 21.4 ± 2.8 years, mean height = 2.01 ± 0.4 cm, mean weight = 88 ± 8.5 kg), power forward (PF) (*n* = 4, mean age, 23.8 ± 4.1 years, mean height = 2.00 ± 0.1 cm, mean weight = 97.3 ± 7.9 kg), and center (C) (*n* = 2, mean age, 31.9 ± 1.5 years, mean height = 2.02 ± 0.2 cm, mean weight = 107 ± 5 kg). It should be noted that some players filled more than one playing position. For instance, certain players transitioned from the SF to the PF within specific plays due to tactical decisions (e.g., foul trouble) by the head coach. In such scenarios, these players were categorized differently, reflecting their positional change during the analyzed moves, as opposed to their initial designated playing position. For the analysis, all situational position changes were considered. Also, not all 19 players could be incorporated into the analysis, because some did not receive playing time and/or were unable to play due to injuries. This led to a refined cohort of 9 players who each participated for an average duration of 10 min across all games. Players were routinely filmed during all games in the course of the competitive season. All players confirmed the usage of video material for analytical purposes by contract and all information was publicly available on a streaming service (i.e., MagentaTV). Each participant provided written consent for participation in the study, which was fully conducted according to the principles of the declaration of Helsinki (46) and approved by the local ethics committee (Grant Number: 2021-30).

### 2.3 Observational procedure

Throughout the 2020–2021 German first league season, a longitudinal video-based analysis was conducted within one professional basketball team participating in this national league by two independent raters, possessing a minimum of 20 years of basketball-specific experience in national and international competition formats (DW) and a minimum of ten years in video analysis and game tagging (JJ). Data collection involved systematically quantifying contact actions of each player across ten home games during the season. All contacts were analyzed in real time and, if necessary, by slow motion or frame-by-frame sequencing. Contacts were included in the analysis only if they had a recognizable impact on the game, as defined by Meehan et al. (47). This included game situations with frequent physical contact resulting from specific basketball movements, such as setting a screen or boxing out during a rebound, or using physical contact to disrupt an opponent's dribble drive, which contain a clear impact among involved players. These scenarios represent frequent game sequences in professional basketball that involve contact but do not constitute targeted collisions. In this context, a distinction was made between recognizable contacts and those in collision sports (e.g., tackling in rugby), where collisions are an integral and expected part of the sport (47). Furthermore, incidental touches, body stripes, and other non-substantive forms of contact (e.g., cheering or substitution), were not considered.

**Figure 1:**
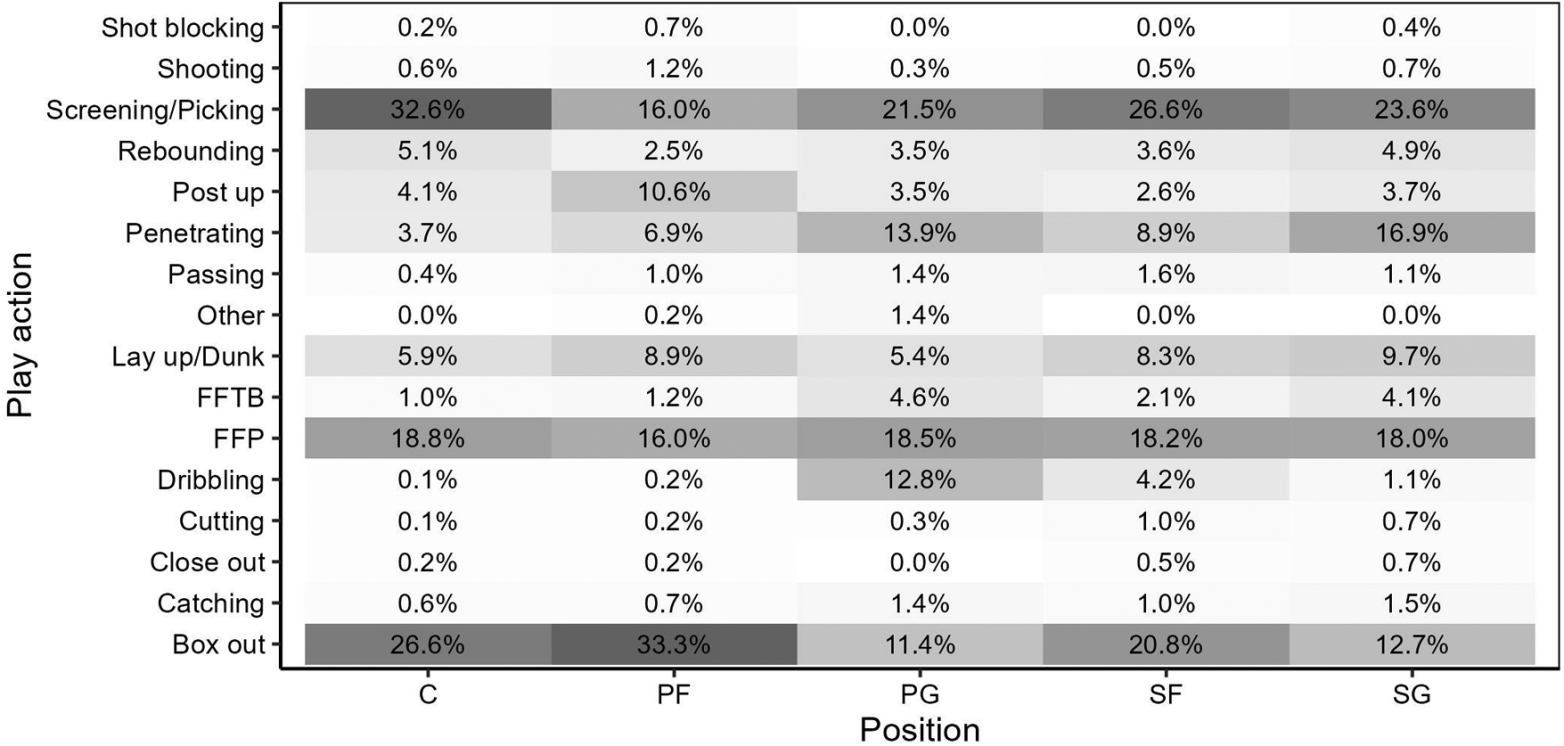
Percentage of play actions containing contacts per playing position. All observed contacts are included. FFTB, fight for the ball; FFP, fight for position.

Preceding the video inspection, an analytical catalogue was formulated by drawing upon insights from prior observational studies in basketball (48). This catalogue comprised seven overarching items encompassing 40 specific factors [see Table 1; for wording, see (48)]. Items I and III classify the positions of players and opponents engaged in contact situations. In cases where there was a change in the opposing player, leading to inconsistent classification, the contact source was categorized as “Other”. Opponents and teammates were identified as contact sources (Item II), whereas “Other” encompassed such contacts as impacts on the floor, court, or basket. Game phases (Item IV) were categorized into four situations covering the majority of basketball scenarios, whereas play actions (Item V) represented various techniques and tactical elements. “Other” play actions included scenarios not clearly assignable to a specific factor (e.g., passes accidentally going into the basket) (see Figure 1). Four body areas (Item VI) were defined as points of contact with lower and upper extremities incorporating specific segments. This broader categorization was chosen because isolated labeling of individual segments was not feasible in some scenarios. This is attributed to rapid multi-contact situations (e.g., knee and lower leg) and low contact counts, especially in distal anatomic segments. Item VII segmented contacts into two factors to analyze kinematic displacements occurring during the contact.

**Table 1:** Items, categories, and factors included for the observation [Modified from (48)].

Item	Category	Factor
I	Playing position	Point guard (PG), shooting guard (SG), small forward (SF), power forward (PF), center (C)
II	Source of contact	Opponent, teammate, other
III	Playing position opponent	vs. PG, vs. SG, vs. SF, vs. PF, vs. C, other
IV	Game phase	Half-court defense, transition defense, fast break, set offense
V	Play action	Box out, catching, close out, cutting, dribbling, fight for position (FFP), fight for the ball (FFTB), lay up/dunk, other, passing, penetrating, post up, rebounding, screening/picking, shooting, shot blocking
VI	Point of contact	head/neck, torso, upper extremity, lower extremity
VII	Form of contact	Kinematic displacement (occurrence of any positional changes due to external contact), no kinematic displacement (absence of positional change despite external contact was made)

To assess the test-retest reliability of the analytical catalogue, a pilot study involving one entire game was conducted. The inter-rater agreement for all items was evaluated over a one-week interval. Using Cohen's kappa (*κ*), the analysis of test-retest-reliability for Rater 1 resulted in “very good” for Item I (*κ* = .98), III (*κ* = .96), IV (*κ* = .97), V (*κ* = .93) VI (*κ* = .88), VII (*κ* = .84) and “good” for Item II (*κ* = .79). Rater 2 showed “very good” (Item I, *κ* = .92; Item III, *κ* = .89; Item IV, *κ* = .89; Item V, *κ* = .92; VI, *κ* = .84) and “good” (Item II *κ* = .78; Item VII *κ* = .78) test-retest-reliability. Inter-rater-agreement resulted in “very good” (Item I, *κ* = .91; Item III, *κ* = .94; Item IV, *κ* = .89; Item V, *κ* = .91; Item VI, *κ* = .82) and “good” (Item II, *κ* = .79; Item VII, *κ* = .74) concordance. For the final analysis, contact actions were identified and labeled using Focus for teams by SBG Sports Software © and subsequently exported to a separate worksheet using Microsoft Excel (Version 2311). Sequences that were unanalyzable due to inadequate visibility (e.g., concealed by teammates or opponents) on the video were further assessed by both raters. In instances in which contacts were not included, the agreement between the raters was examined. For the final determination, all scenarios were discussed by both raters until an agreement was reached (49).

### 2.4 Statistical analysis

To assess observational consistency, the agreement between the two raters for each contact sequence was quantified using *κ*. Interrater agreement was assessed for each factor listed in Table 1. These individual values were aggregated to calculate the mean values of the individual items. Threshold values for *κ* were classified as follows: <.2 (poor), .2–.4 (fair), .4–.6 (moderate), .6–.8 (good), and .8–.0 (very good) (50). The exploratory data analysis regarding the positional contact count is presented in means and standard deviations. Leven's test for homogeneity of variances (*p* = .1), and Shapiro-Wilk test (*p* = .06) showed non-significant results, confirming equal variances and normal distribution of the data. Subsequently, a one-way analysis of variance (ANOVA) was conducted to determine significant differences in contact count among different positions. Eta squared (*η*²) was used as a measure of effect size, with thresholds defined by Cohen (51): small (.01), medium (.06), and large (.14). *Post hoc* comparisons were conducted using the Tukey Honest Significant Difference (HSD) test to examine specific pairwise differences. Playing-position-specific variations concerning Items II–VII (Table 1) were examined utilizing the chi-square test of association and the Fisher exact test with a *post hoc* analysis incorporating standardized residuals (52). The significance level for all statistical tests were set at *p* < .05. All graphics and statistical analysis were performed using RStudio software ® (Version 4.3.3).

## 3 Results

**Figure 2:**
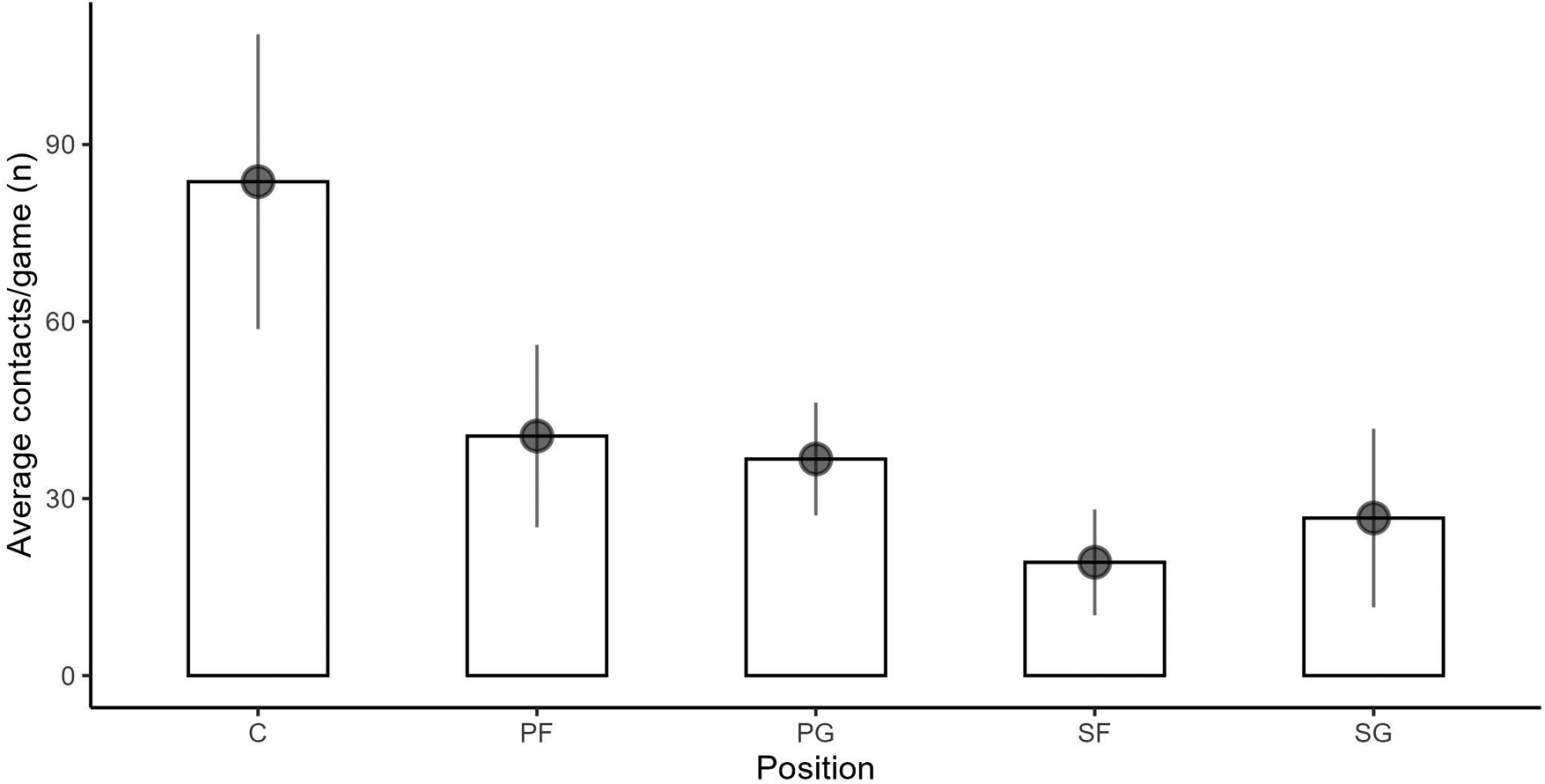
Positional average contact count per game over the course of analyzed games. Data are presented as mean values and standard deviation.

For the ten games the agreement level between both raters could be defined as “very good” for Items I (*κ* = 0.98), II (*κ* = 0.96), IV (*κ* = 0.89), and V (*κ* = 0.91). A level of agreement ranging from “good” to “moderate” was observed for Items III (*κ* = 0.77), VI (*κ* = 0.79), and VII (*κ* = 0.58). Out of 2,079 contacts, a total of 10 contacts with a “good” agreement level (*κ* = 0.78) could not be identified adequately due to limited visual inspection, resulting in a final 2,069 contacts being included across the ten games. The C (*n* = 837, 40.5% of total) received a higher number of contacts than PF (*n* = 406, 19.6% of total), PG (*n* = 367, 17.7% of total), SG (*n* = 267, 12.9% of total) and SF (*n* = 192, 9.3% of total). The mean contact count per position across all games is displayed in Figure 2. ANOVA revealed a significant effect of player position on contact frequency *F*(4, 45) = 24.95, *p* < .001, *η*² = .69. Post-hoc comparisons showed significant differences in contact among between PF-C, PG-C, SF-C, SG-C (all *p* < .001) and SF-PF (*p* < .005). The C (*n* = 837, 40.5% of total) received a higher number of contacts than PF (*n* = 406, 19.6% of total), PG (*n* = 367, 17.7% of total), SG (*n* = 267, 12.9% of total) and SF (*n* = 192, 9.3% of total). The Fisher exact test and the chi-square test revealed significant associations between the player's position and the source of contact [*χ*²_(8, 2,069)_ = 15.6, *p* = .048], the opponent's playing position [*χ*²_(20, 2,069)_ = 301, *p* < .001], the game phase [*χ*²_(12, 2,069)_ = 175, *p* < .001], the play action [*χ*²_(60, 2,069)_ = 421, *p* < .001], and the point of contact [*χ*²_(12, 2,069)_ = 67.2, *p* < .001]. No significant association was found regarding the form of contact [*χ*²_(4, 2,069)_ = 6.94, *p* = .139].

**Figure 3:**
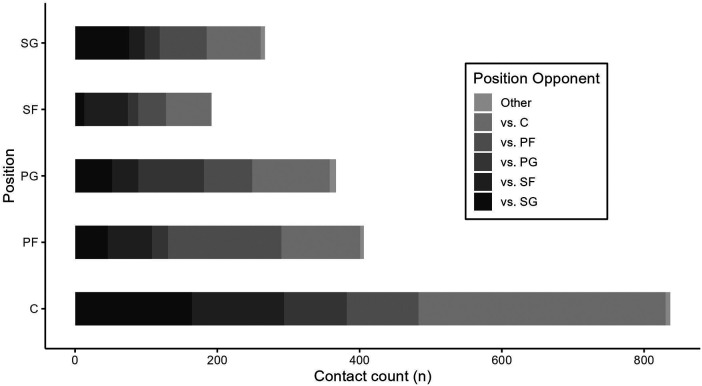
Contact frequency relative to the oppositional position. Data are presented on all contacts for each position.

Overall, the predominant source of contacts was made by opponent players (97.8%). Falls or contacts with objects on the court (“Other”) ranked as the second most frequent (1.5%), except for SF. Contacts initiated by teammates were the least frequent across all playing positions (0.7%). Figure 3 presents an overview of the opponent's playing position during contact. Interestingly, the highest residual associations for each playing position were identified for the corresponding opposing counterpart (C = 5.89, PF = 10.29, PG = 8.81, SF = 6.8, SG = 5.38, all *p* < .05). With regard to the observed game phases, the majority of contacts were observed during half-court defense (46.1%) and set offense (48.9%). Fast breaks (2.9%) and transitional defensive phases (2.2%) of the game exhibited significantly fewer contacts between players. Positional *post hoc* analyses utilizing standardized residuals indicated the most contacts for the C during half-court defense (−9.26), set offense (10.84), and transition defense (−2.51), whereas the SG had significantly more contacts on fast breaks (6.01).

The observation of different play actions showed that it was predominantly screening/picking (*n* = 531, 25.7%), box outs (*n* = 474, 22.9%), and fights for position (FFP) (*n* = 373, 18%) that led to physical contact, whereas close outs, shot blocking, while “Other” actions (all *n* = 6, 0.3%) led to contact less frequently. Figure 1 presents a visualization of the relative positional distribution in the aforementioned defined play actions. The visualization indicates that contacts were most frequent during screening/picking, box outs, and FFP. In contrast, the fewest contacts were observed during cutting movements, shot blocking, and close outs. *Post hoc* analyses and the percentile distribution indicate significant associations between C (32,6%, 5.93) and PG (21.5%, −1.98) contact involvement during screening/picking. Conversely, even though SF and SG had the highest percentage involvement in screening/picking, there was a statistical divergence. Standardized residuals indicated that SF were more likely to experience contact during post ups (−1.62), whereas SG had significantly more contacts during penetration to the basket (5.36). PF also exhibited a deviation between observed and expected values. Whereas they received a higher percentage of contacts during box outs, the standardized residuals, similar to SF, were highest in post ups (5.61).

**Figure 4:**
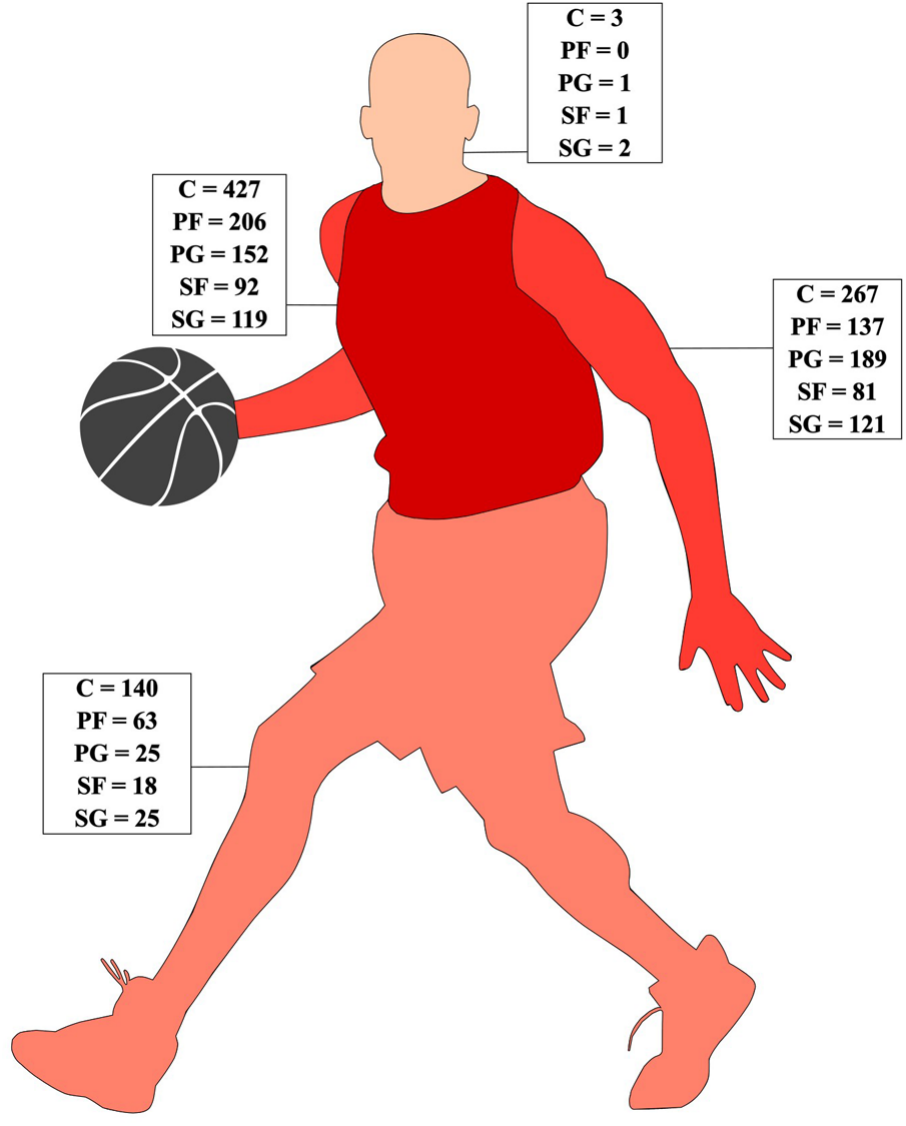
Positional distribution of contacts across body areas. A deeper shade of red indicates a higher frequency of contacts.

Regarding contact points, the torso was the most frequently affected area (*n* = 996, 48.1%), followed by the upper (*n* = 795, 38.4%) and lower extremities (*n* = 271, 13.1%), whereas the head/neck (*n* = 7, 0.3%) were the least impacted. Figure 4 displays playing position-specific distributions of the contact points. Positional *post hoc* analysis showed that C and PG had significantly more contacts at the arms (−4.95; 5.56), torso (2.4; −2.95), and legs (4.07; −3.97). Sustained contacts at PF (−2.24) and SG (2.55) showed a significant association with the upper extremities. SF showed no significant differences in terms of contact points. Furthermore, a total of 81.4% (*n* = 1,684) of all contacts resulted in a kinematic displacement, whereas the remaining 18.6% (*n* = 385) exhibited no change in playing position during the physical contacts between players.

## 4 Discussion

The primary objective of this investigation was to conduct a video-based analysis of physical contacts during professional male basketball games. As highlighted by previous research emphasizing the importance of considering contacts during gameplay (28), our results support the assumption that contacts among players are prevalent across all playing positions in basketball (Figure 2). Notably, the center (C) position emerged as the recipient of the highest frequency of contacts throughout all ten games with over 40% of the total number of contacts. This outcome aligns with findings reported by Ibáñez et al. (53) and Ribeiro et al. (54). It should be mentioned that a basketball player's position is influenced predominantly by individual factors such as basketball-specific skills, body height, and body mass, as highlighted by Puente et al. (28) and Svilar et al. (43). In conjunction with these considerations, a possible explanation for the increased contact experienced by C may be attributed to their tactical role that often requires them to occupy smaller spaces around the basket. These positional demands for C are confirmed by our results, showing high numbers of box outs (26.6%) and FFP (18.8%), which typically occur close to the basket. Studies by Schelling and Torres (13) and Vanderlei et al. (55) posit that C assume responsibility for shots within the key area by engaging in disputes for both defensive and offensive rebounds and executing forceful maneuvers when competing for spatial dominance (55). A similar explanation is given by Ferioli et al. (8) positing that C gain possession of the ball by executing rapid and intense movements such as offensive maneuvers to score or secure rebounds. Current studies indicate that C experience a lower physical and physiological demand in terms of overall running movements, accelerations, and decelerations (8, 13, 32). However, there is still a lack of evidence regarding the impact of such contact on external and internal loads in the context of basketball.

In addition, within this cohort, results indicate that each playing position engages predominantly in contacts with its corresponding opposing counterparts. However, there are instances of contact in different playing positions. One plausible explanation for this could be mismatches or tactical maneuvers such as intentional physical interactions during offensive plays. Notably, an exception to this trend is observed in the case of the point guard (PG), who exhibits comparable physical contact frequencies with the C. This pattern may be explained by the strategic deployment of ball screens, a significant facet of gameplay, utilized in most offensive plays in professional basketball (56–58). Our study has shown similar results, indicating a high number contacts during ball screens for all positions (Figure 1). The setting of ball screens involves a collaboration between two players, typically an inside player as the screener and an outside player as the beneficiary (56). This strategic maneuver is defined as a fundamental technical–tactical element wherein the screener executes a screen to create a favorable situation and advantage for the player with possession of the ball. This advantageous position is sought for purposes of passing, shooting, or penetrating to the basket (42, 57, 59). The unique dynamic introduced by ball screens may contribute to the atypical equalization of contact between PG and C in contrast to the prevailing pattern observed among other players.

The majority of contacts in this study were sustained during set offense and set defense, aligning with the findings of Achenbach et al. (48). This pattern suggests that contacts are predominantly employed in organized situations such as set plays. Concurrently, this trend is reflected in in-game situations in which contact is utilized such as screening/picking, box out, or FFP (Figure 1). Furthermore, results reveal that, within this cohort, these three game actions (screening/picking, box out, and FFP) constitute the most prevalent contact actions across all five playing positions. Ribeiro et al. (54) indicated that the PG experiences the highest frequency of contacts. Regarding contact with other players, Puente et al. (28) and Ibáñez et al. (60) reported body impacts (including physical contacts) exceeding 5 g per minute (Sum of impacts measured in g-forces in the three planes per minute). Whereas it is acknowledged that contact in basketball can lead to injuries necessitating players break, as discussed by Achenbach et al. (48), Brumitt et al. (61), and Minghelli et al. (62), it is crucial to recognize that physical contact is inherent to basketball, constituting an integral aspect of the game that does not invariably result in severe injuries.

However, existing research on the impact of physical contact on internal load responses has primarily focused on collision sports such as rugby (63–65). Consequently, the direct applicability of these studies to basketball in particular is limited. Doeven et al. (66), reports a recovery time on the neuromuscular level up to 48 h after a game which can be explained by the high number of intensive activities (e.g., jumping, shuffling, running) performed during basketball matches (3). Furthermore, for a comprehensive understanding of recovery processes (67), mentioned that various contextual factors need to be considered (e.g., travel duration, individual chronotype, playing style). In this context, the quantification of load induced by physical contacts might be helpful in implementing primary (e.g., nutrition, sleep and rest) and secondary (e.g., supplementation, physical recovery, therapeutic interventions) recovery strategies (68). Also, the implementation of subjective load measures (e.g., differential rated perceived exertion, dRPE) might enhance the understanding of different dimensions of physical efforts such as contacts received (11). A detailed investigation of physical contacts during basketball gameplay could extend the knowledge of internal load responses, building on existing research on inflammatory processes (69), salivary markers (70), and neuromuscular performance (71). Therefore, following a multimodal approach in players’ recovery, the monitoring of load produced by physical contacts in games and practices needs to be considered besides commonly used external load markers in elite basketball.

Our study showed a high number of contacts to the torso and upper limbs, suggesting that these areas experience significant internal load, which may lead to structural reactions such as contusions, tears, impacts, or laceration injuries (72–75). Visual inspection indicated that contacts in the chest-shoulder area (e.g., post ups, screens) are associated with high impacts. This allows us to speculate that especially C and PF, who are frequently involved in these situations, experience higher internal load responses due to physical contacts. Recognizing that controlled studies of muscular responses during game observations are challenging, isolated studies with high internal validity (for an experimental design, see (76) on muscles at different contractile characteristics could provide valuable insights into the impact of physical contact at the muscular level in basketball. Although our analysis revealed a low incidence of head impacts, it is crucial to recognize that contact in sports can result in both musculoskeletal injuries and brain effects. Sports-related concussions, caused by direct blows to the head, neck, or body, expose the brain to impulsive forces during sporting activities (77). Repeated concussions pose a risk to long-term brain health (77, 78), and there is concern that even frequent low-level impacts in contact sports can harm healthy individuals. Studies suggest that repeated subconcussive impacts can lead to neurophysiological changes (79–81), emphasizing the importance of monitoring physical contact received in the head/neck area in basketball.

On the other hand, diverse contact situations are evident, with an elevated incidence of contact for the PG during dribble situations and for the PG, SG, and SF when penetrating to the basket. Additionally, the augmented contact observed for the PF during post ups warrants attention. Indeed, distinct playing positions yield disparate outcomes in terms of contact scenarios, indicating the imperative to consider the specificity associated with each playing position. Even among players occupying the same playing position, differences in on-court functions may manifest. Another compelling indication supporting the need for distinct consideration of playing positions is given by the distribution of contact points on the body. In this context, C consistently exhibit the highest frequency of contacts across all anatomical areas. Delextrat et al. (82) offer insights into these phenomena, characterizing inside players as engaging predominantly in static efforts such as blocking and positioning for rebounds. This underscores the need to recognize and analyze playing positions individually, acknowledging the diverse roles and demands inherent to each playing position on the basketball court.

The monitoring of the contacts players experience could potentially mitigate the risk of injury. Similar to the monitoring of in-game workload, gaining insights into in-game contact loads can empower coaches to formulate more targeted training and recovery strategies, enhancing the overall preparation of their players (83). This information underscores that determining the physical load required for competitive basketball cannot rely solely on measuring the quantity and intensity of dynamic actions. Acknowledging the limitations of relying solely on dynamic metrics, the inclusion of physical contact monitoring provides a more comprehensive understanding of the physical demands placed on players during a basketball game. By integrating information on both dynamic actions and contact loads, coaches can tailor training regimens and recovery strategies more effectively and contribute to optimizing player performance and injury prevention.

### 4.1 Practical implications

This study advances the understanding of physical contacts in professional basketball and highlights the importance of considering physical contacts when assessing internal load after gameplay. Utilizing video-based observation in a professional basketball setting, the study provides initial insights into the contextual characteristics of physical contacts across various playing positions. These analyses need to be deepened and extended in future research. For practitioners, the findings offer valuable information for conceptualizing load in training, conditioning, and recovery strategies for basketball players. Notably, the examination of players’ physical contact profiles during gameplay reveals significant discrepancies across individual playing positions. The increased number of contacts observed across playing positions underscores the need for tailored resistance training regimes to address the distinct demands encountered by players in different roles. From a training perspective, it is imperative to expose players to manageable levels of contact, isometric exercises, and eccentric loads on a regular basis. This approach aims to minimize muscle damage and facilitate adaptive responses to the specific demands of basketball. Particularly noteworthy is the emphasis on the fatiguing effect of contusions, as demonstrated by Barnes et al. (76). Their study, utilizing an unspecific experimental contusion model, suggests that the impact forces experienced are comparable to, or slightly lower than, those observed in contact sports such as rugby union tackles (∼1,600–2,000 N) or martial art kicks (∼1,500–2,000 N). Consequently, the authors speculate that the physiological responses observed are indicative of those typically associated with sport-related contusion injuries. Although direct cross-sport comparability presents challenges, these findings offer a preliminary framework for initiating contusion monitoring in basketball, with the aim of broadening load monitoring practices within the sport. Moreover, Barnes et al. (76) highlight that contusions share similarities with eccentric muscle injuries in certain aspects, underscoring the relevance of these findings to the broader context of sports medicine.

Furthermore, basketball coaches can leverage the insights provided by our research on players' contact demands during different game phases to tailor individualized and team-based training sessions. Specifically, exercises for C, given the heightened contact demands inherent to this playing position, should emphasize the development of specific movements, body contacts, and collision scenarios. Guards, who frequently navigate ball possession amidst diverse contact situations, would benefit from dedicating substantial time to a variety of ball-related exercises aimed at enhancing their skills in such contexts. Another consideration arises when players operate across multiple playing positions, whether for tactical or strategic reasons or because modern basketball teams often deviate from strict adherence to traditional playing position classification systems. In these instances, the load profile for these players becomes inherently more complex, potentially affecting the physical demands across various playing positions and individuals (23, 84). Consequently, it is essential to exercise caution when applying the results pertaining to physical demands classified by playing positions in a practical setting, particularly in cases where the categorization and clarification of playing position roles within the team are ambiguous.

### 4.2 Limitations

Match video analysis is an indispensable tool in sports, particularly for training and coaching purposes. However, its efficacy is contingent upon various factors and relies primarily on image quality, resolution, and available camera angles. Whereas video analysis offers invaluable insights, its utility is not without limitations. Accessibility to all contacts via video analysis is not assured. Challenges arise when attempting to identify precise contact events due to occurrences being outside the camera frame or obscured by players or referees. Blind spots, created by players or equipment (e.g., basket), further impede the accurate identification of critical events. Limited camera positions exacerbate these challenges by restricting visibility of certain pitch areas, potentially resulting in information gaps—particularly during pivotal event moments beyond the field of vision.

It should also be acknowledged that our study identifies only the initial contact, which often encompasses several ranges. Consequently, our findings do not account for more detailed analyses of body areas. Future research efforts could delve deeper into these nuances to elucidate the intricacies of physical contacts in basketball more comprehensively. Lastly, the authors acknowledge that a G*Power analysis is typically conducted to determine the appropriate sample size for detecting significant effects. However, in this case, a power analysis was not performed due to the exploratory and descriptive nature of the study, which aimed to provide initial insights and preliminary observations into this research phenomenon.

### 4.3 Future directions

It is important to note that our study focuses solely on elite male basketball players from a single country, potentially limiting the generalizability of our findings across genders, cultures, playing levels, and nations. Thus, there is a critical need for further research to bridge these gaps and provide a more comprehensive understanding of contact dynamics in basketball across diverse contexts. Furthermore, as a multifaceted team sport, basketball encompasses dynamically interconnected game events and situations (85). Studies have analyzed situational variables such as game location, match status, and opponent quality in order to explore their impact on performance metrics (30). Disparities in fundamental player characteristics, including physical and physiological traits, between top and bottom teams contribute to variations in attacking and defensive contacts. Although not within the scope of our study, future research should comprehensively investigate this aspect.

Whereas our study utilized video analysis to identify player contacts, it is important to acknowledge that quantifying the associated load resulting from these contacts cannot be accomplished solely through video analysis. Video analysis provides valuable visual data on the occurrence and nature of contacts during gameplay. However, it lacks the capacity to measure the physiological or biomechanical impacts of these contacts on players directly. Micro-technical devices such as LPS or GPS offer a potential supplementary method for quantifying the load resulting from player contacts during basketball gameplay in future research. Previous studies have demonstrated the utility of accelerometers or specific load metrics in describing body contacts and assessing physical loads encountered by athletes across different sport types (86–88). In addition to video analysis and micro-technical devices, utilizing psychological measures such as ratings of perceived exertion or physiological measures such as heart rate variability offer other valuable methods for quantifying the internal load response resulting from player contacts (89). Furthermore, future examinations of intra- and interindividual variabilities in fatigue markers (e.g., creatine kinase or urea) could enhance the understanding of physical contacts on the dynamics of internal load responses. Implementing such methods provides a valuable complement to the video-based quantification of physical load, enhancing the understanding of the holistic impact of player contacts on athletes' performance and well-being in professional basketball.

## 5 Conclusion

The results of our study underscore the assumption that basketball cannot be considered as a noncontact sport as initially developed by Naismith. Given their frequent occurrence across various play actions and game phases during competitive matches, incorporating physical contacts into analyses seems appropriate in order to assess external and internal load in professional basketball. Additionally, our analysis highlights that contacts affect different anatomical regions of basketball players. Thus, our findings emphasize the complexity of physical contacts in shaping the overall load profile of professional basketball players. In summary, the results suggest that future research should consider incorporating physical contact in the assessment of physical load in basketball in order to gain a more comprehensive picture of external load and internal load responses. By acknowledging the significance of these contacts, researchers and sports practitioners can better understand the holistic impact of player interactions on physiological and biomechanical demands, and this will lead ultimately to more effective training strategies and injury prevention measures in basketball.

## Data Availability

The raw data supporting the conclusions of this article will be made available by the authors, without undue reservation.
